# Potentiated lung adenocarcinoma (LUAD) cell growth, migration and invasion by lncRNA DARS-AS1 via miR-188-5p/ KLF12 axis

**DOI:** 10.18632/aging.203632

**Published:** 2021-10-13

**Authors:** Yangyang Liu, Lu Liang, Liang Ji, Fuquan Zhang, Donglai Chen, Shanzhou Duan, Hao Shen, Yao Liang, Yongbing Chen

**Affiliations:** 1 Department of Thoracic Surgery, The Second Affiliated Hospital of Soochow University, Suzhou, China; 2 Department of General Surgery, The Affiliated Zhangjiagang Hospital of Soochow University, Suzhou, China; 3 Department of Thoracic Surgery, Shanghai Pulmonary Hospital, Tongji University, School of Medicine, Shanghai, China

**Keywords:** lung adenocarcinoma, lncRNA, DARS-AS1, miR-188-5p, Krüppel-like factor 12

## Abstract

Lung adenocarcinoma (LUAD) is the most common histological type of non-small cell lung cancer (NSCLC). Due to the nonspecific early symptoms, the majority of the diagnosed LUAD patients are in the middle and late stages, with multiple metastases, and have missed the optimal period for treatment. Current studies have reported lncRNA DARS-AS1 as a cancer-promoting gene that expedites tumorigenesis. This is the first study demonstrating that DARS-AS1 is involved in the mediating process of LUAD. Cell functional experiments revealed that lncRNA DARS-AS1 participated in enhancing LUAD proliferation, invasion, and migration by inhibiting miR-188-5p. The investigation on DARS-AS1/miR-188-5p led to the discovery of KLF12 as a downstream target of miR-188-5p, and the regulatory pathway was established as DARS-AS1/miR-188-5p/KLF12. According to western blot results, DARS-AS1 promoted LUAD cell growth, migration, and invasion via stimulation of the PI3K/AKT pathway, activating the EMT process, and up-regulating the CyclinD1 and Bcl-2 proteins. This was the first report on the DARS-AS1/miR-188-5p/KLF12 axis and offered a novel strategy for early diagnosis, a new therapeutic method, and an improved prognosis for LUAD.

## INTRODUCTION

Lung cancer is responsible for over 27% of cancer-related mortalities worldwide, with non-small cell lung cancer (NSCLC) accounting for 80% of the cases [1, 2]. Lung adenocarcinoma (LUAD) is the most common histological type of NSCLC. Despite significant progress in lung adenocarcinoma therapeutic methods, including surgical treatment, target therapy, and early cancer detection, the overall LUAD survival rate remains low [3]. Currently, cytology screening and imaging examination are sensitive cancer screening tools, but they are not effective for the early detection of lung adenocarcinoma [4, 5]. Furthermore, more than half of the patients are still unable to benefit from targeted therapy in a narrow sense [6, 7]. Hence, the development of early diagnostic biomarkers and prognostic biomarkers is essential for the accurate prediction of clinical outcomes and early detection of lung adenocarcinoma.

Long non-coding RNA (lncRNA) is a type of RNA that has a length of over 200 nt but lacks the ability to encode proteins, which plays a considerable role in regulating chromatin dynamics, gene expression, growth, differentiation, and development [8]. LncRNA has different expression patterns in specific cells, tissues, and time points. It regulates cell proliferation, differentiation, stem cell reprogramming, tumorigenesis, and drug resistance by directly interacting with DNA, mRNA, or protein to modulate chromatin modification or structure, transcription, splicing, and translation [9–11]. For decades, lncRNA has been found to express its characteristics in different malignant tumors, but its mechanism of regulating the occurrence, proliferation, metastasis, drug resistance, apoptosis, and other physiological and pathological processes of specific malignant tumors remains in the early stages of research. Therefore, there is much focus on examining how lncRNA regulates signal pathways to affect LUAD.

Recently, accumulating evidence has confirmed that the lncRNA DARS-AS1 is a cancer-promoting gene that expedites tumorigenesis of multifarious types of malignant cancers. Huang et al. identified DARS-AS1 as a tumor-promoting factor in mediating ovarian cancer by directly acting as a sponger of miR-532-3p [12]. According to Jiao et al., DARS-AS1 was a cancer-promoting gene that silenced miR-194-5p in clear cell renal cell carcinoma [13]. Similarly, many studies have confirmed the tumor attenuating effect of DARS-AS1 in various types of cancers, including myeloma [14] and thyroid cancer [15]. Nevertheless, little is known regarding the exact effect of lncRNA DARS-AS1 in LUAD, prompting us to investigate it further.

MicroRNAs (miRNAs) are endogenous, single-stranded, small noncoding RNAs that have a length of roughly 18–25 nucleotides [16]. Current studies have verified that microRNAs (miRNAs) are useful biomarkers for biological and clinical characteristics [17], and aberrant miRNA expression is involved in the proliferation, differentiation, apoptosis, and all basic cell processes related to carcinogenesis [18]. Studies have shown miRNA to be potential biomarkers for cancer diagnosis, prognosis evaluation, and treatment targets. Some specific miRNAs, such as mir-432, mir-210, miR-145, and miR-31, can predict the clinical outcome of patients with lung adenocarcinoma [19]. The aberrant expression level of miR-188-5p in cancers has gained attention due to its regulatory role in the tumor development process. MiR-188-5p was identified as a biomarker for the prognosis of acute myeloid leukemia [20]. In addition, it has an inhibitory effect on prostate cancer growth and migration [21]. Furthermore, many studies have also confirmed the participation of miR-188-5p in different types of carcinomas, including keloids [22], breast cancer [23], retinoblastoma [24], gastric cancer [25], and hepatocellular carcinoma [26]. However, there is limited understanding of the specific effect of miR-188-5p in LUAD. This study aimed at discovering novel diagnostic and prognostic biomarkers for LUAD, as well as developing a promising novel therapeutic strategy.

## MATERIALS AND METHODS

### Bioinformatics prediction by TCGA database

For bioinformatics prediction of LUAD, TCGA database was applied (https://www.cancer.gov/) for lncRNA expression forecast. Threshold values set as *p* < 0.05 and |log FC| > 1.

### Patients’ specimens and cell lines

Altogether fifty pairs of LUAD specimens and normal lung tissues were resected from LUAD patients in department of thoracic surgery, the second affiliated hospital of Soochow University. The extracted tissues were immediately frozen by liquid nitrogen and were subsequently kept in cold storage under −80°C till experiments. All related patients were informed about the investigation and signed informed consent letter. The experiment was approved by the Ethics Committee of The Second Affiliated Hospital of Soochow University. For the incubation of the cell lines in this study, human bronchial epithelial cells HBE cell line and lung adenocarcinoma (LUAD) cell lines (NCI-H23, A549, HCC827, PC-9 and C422L) were cultured with DMEM medium which was also supplemented with 10% FBS under a condition of 37°C and 5% CO_2_.

### Cell transfection

Small interfering RNA against DARS-AS1 (si-DARS-AS1) and small interfering RNA negative control (siRNA) were applied for the knockdown of DARS-AS1 in LUAD cell line A549. While full length of DARS-AS1 (DARS-AS1 ov) and related negative control were used for up-regulating DARS-AS1 in PC-9 cell line. MiR-188-5p inhibitor and mimics were adopted to organize the expression level of miR-188-5p. Full length of KLF12 (KLF12 ov) and related negative control were used for up-regulating KLF12 in A549 cell line. All mentioned control factors were then transfected into cells via Lipofectamine^™^ 2000 adhered to the provided protocol.

### Quantitative reverse transcriptase polymerase chain reaction (qRT-PCR)

RNA extraction was conducted with TRIzol reagent. Then the RNA was reverse-transcribed into cDNA. The expression of lncRNA DARS-AS1 and KLF12 was detected by ABI 7900HT RealTime PCR System using SYBR Green assays and GAPDH was used as the internal control. The expression of miR-188-5p was measured using TaqMan MicroRNA Assays and U6 was treated as an internal control. 2^−ΔΔCt^ method was applied for the quantification.

### Cell counting kit-8 assay for cell growth evaluation

Cells with different transfection were transferred into 96-well plate and were allowed to attach to the well and grown for 24, 48, 72 and 96 h. Thereafter, CCK-8 reagent was added into each well with an amount of 20 μL, which following 2 h of cell incubation under 37°C. Finally, determine the absorbance at optical density (OD) of 450 nm by microplate reader.

### BrdU assay

10 μM of BrdU was added into the investigated cells and waiting for 0.5–1 h of incubation. Then cells were resuspended at 4°C and frozen in 70% V/V ethanol. Centrifuge the supernatant and wash using PBS, the cells were cultured at ambient temperature using HCl (2 M) for 0.5 h. After PBS washing twice, the anti-BrdU antibody were given to the suspension and the treated cells were cultured avoid light for 20 min. Subsequently, 100 mg/ml of RNAse were used for 15 min and following FCM analysis for the detection of cell growth capacity.

### Colony-forming assay

The cells in logarithmic growth phase were taken to prepare cell suspension, which was then diluted by gradient multiple and inoculated into the dish containing the culture medium for 2–3 weeks. Frequently observed, when the visible clone appeared in the culture dish, the culture was terminated. Discard the supernatant, fix with 4% paraformaldehyde, and dye with Gimsa for 10–30 min. The clones were photographed and counted. The experiment was repeated three times.

### Evaluation of cell migration and invasion capacity from transwell assay

The cells were seeded on 6-well plates, confluence about 80%, and transfected. Then the pores used for invasion experiments should be coated with matrix adhesive. Matrigel was diluted with DMEM (FBS free) and added into Transwell upper chamber at 60 μL/well. Cells were digested and counted about 12 hours after transfection. The concentration of cells was adjusted to 1.5 × 10^5^/ml in DMEM medium containing 1% FBS. 100 μL was laid on the adhesive surface at the bottom of upper chamber or directly into the bottom of upper chamber. Add 600 μL DMEM (20% FBS) into the lower chamber. Check under the 24 well plate and put it into the cell incubator after confirming that there is no bubble. After 48 hours, the cells in the 24 well plate were removed, washed with PBS for 3 times, and fixed at 4°C with 500 μL / well methanol for 1 h. Take out the chamber to dry, during which methanol is recovered and dried. Dye with 0.5% crystal violet for 1 h. PBS was used to wash for 3 times, the upper chamber was gently wiped with cotton swab, and the small chamber was air dried. The cells penetrating into the lower layer of the upper chamber were observed and counted in 6 random fields.

### Flow cytometry

Cells in logarithmic growth phase were inoculated in a 60 mm diameter culture dish with 1 × 10^4^ concentration per well, cultured for 48 h. After normal digestion, the cells were washed twice with PBS, and then re suspended in 500 μL binding buffer, mixed with 5 μL Annexin V-FITC and 5 μL PI, and stained in dark at room temperature for 15 min. The apoptosis rate was detected by flow cytometry (FCM, BD FACSCalibur), The upper left quadrant means (Annexin V-FITC)^−^/PI^+^, indicating necrotic cells; The upper right quadrant means (Annexin V-FITC)^+^/PI^+^, indicating late-apoptotic cells; The lower right quadrant means (Annexin V-FITC)^+^/PI^−^, indicating early-apoptotic cells; The lower left quadrant means (Annexin V-FITC)^−^/PI^−^, indicating living cells.

### Subcutaneous tumor transplantation

5 × 10^5^ / ml LUAD cells suspension was inoculated into the right armpit of mice. Each mouse was inoculated with 0.2 ml of tumor cell suspension by subcutaneous injection. After inoculation, the general condition of experimental animals in each group was observed, and the tumor growth of mice in each group was observed. From the 3^rd^ day of inoculation, vernier caliper was used to measure the long diameter and short diameter of tumor in the tumor bearing group every 3 days, and the tumor volume (v = 1/2ab^2^, v represents the tumor volume, a represents the tumor long diameter, b represents the short diameter of the tumor), the tumor size of each group of mice is compared, and the tumor growth curve is drawn. And the average tumor weight was measured after executing mice and extracting tumor tissues until 3 weeks after the injection. Animal experiments were approved by the Animal Ethics Committee of The Second Affiliated Hospital of Soochow University.

### Orthotopic tumor transplantation

To establish orthotopic xenografts, A549 cell transfected with si-DARS-AS1 or si-NC and PC-9 cell transfected with DARS-AS1 ov or NC were harvested by treatment with trypsin/EDTA, washed with cold PBS by centrifugation, and then resuspended in PBS/matrigel and kept on ice before used. The mice were anesthetized with sodium pentobarbital, a 5 mm incision was made on dorsal side over left lung, about 1 cm above the lower rib line, fat and muscles were separated to visualize lung movement. 1 × 10^6^ tumor cells were injected into left lung parenchyma directly at the depth of 3 mm. The wound was closed with suture. mice were dissected on day 21 to determine the growth of the orthotopic tumors at the site of injection and the extent of right lung and thoracic metastasis.

### Dual-luciferase activity evaluation

LUAD cells A549 were seeded into 24-well plate and were allowed to reach 70% confluence. Each well was transfected with 100 ng luciferase vector, 400 ng mir-188-5p mimics or mimics NC or other plasmids (pGL3-DARS-AS1-wt, pGL3-DARS-AS1 mut, pGL3-KLF12-wt and pGL3-KLF12-mut). After 24 to 48 hours of transfection, the medium was discarded and PBS was washed twice. The lysate attached to dual luciferase reporter assay kit was added to lysate cells, and 150 μL was added to each well. The cell lysate was collected and 30 μL was put into the special black enzyme labeled hole. The LARII detection buffer in the dual luciferase reporter assay kit was added to each hole for luminescence detection. The detection termination solution in dual luciferase reporter assay kit was added to each well, and then the luminescence detection was performed again after mixing. The values are derived and calculated.

### Western blot assay

Protein was extracted with RIPA protein solubilization buffer, and protein quantification was performed with BCA kit. Block with 5% skim milk at ambient tempreture for 1.5 h. Corresponding primary antibodies were added (KLF12, p-PI3K, t-PI3K, p-AKT, t-AKT, Bcl-2, vimentin, cyclinD1 and GAPDH) at 4°C Incubate overnight, wash with PBS 3 times, 5 minutes each; secondary antibody was diluted (1:2000) and given to strap, incubate at 25°C for 2 hours, and then use ECL luminescence solution in a dark room Develop and image with ChemiDocXRS+ system.

### Statistical analyses

Statistical analysis was performed with spss 20.0 software and graphpad7, and the measurement data was expressed as mean ± standard deviation. *T* test was used for comparison between the two groups, and one-way analysis of variance was used for comparison between groups. Each experiment was duplicated for at least three times. Kaplan-Meier and log-rank analysis were used for survival analysis. *P* < 0.05 indicates that the difference is statistically significant.

## RESULTS

### Overexpression of DARS-AS1 was examined in lung adenocarcinoma (LUAD) tissues and cell lines

**Figure 1:**
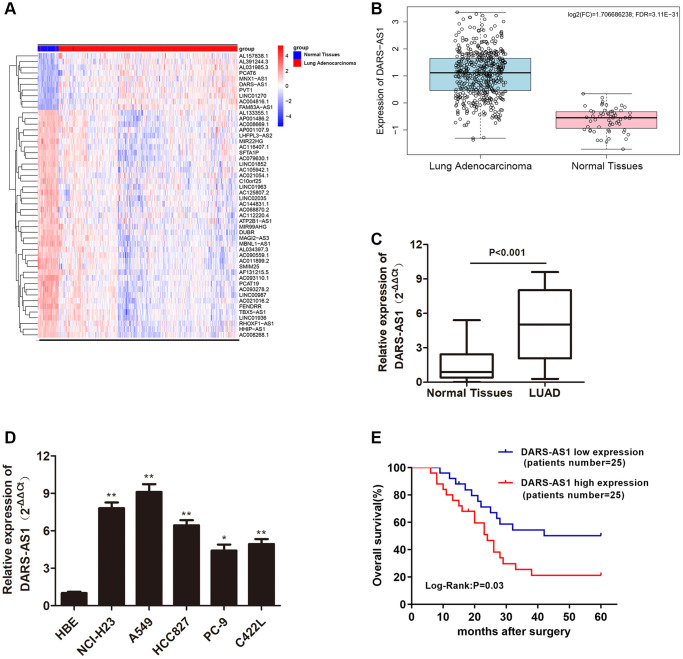
**LncRNA DARS-AS1 was highly expressed in LUAD tissues and cell lines.** (**A**) The top 50 differential expression lncRNA of LUAD identified by a bioinformatics screen in the TCGA database. (**B**) Expression of lncRNA DARS-AS1 was higher than usual in LUAD tissues from the TCGA database. (**C**) Higher expression of DARS-AS1 was identified in 50 LUAD tissues compared to normal tissues (*p* < 0.001). (**D**) Higher expression of DARS-AS1 was identified in LUAD cell lines compared to the HBE cell line (*p* < 0.01). (**E**) High expression of DARS-AS1 in LUAD patients was associated with a poor prognosis (Log-Rank: *p* = 0.03).

According to our preliminary literature research, DARS-AS1 serves as a cancer-promoting gene in various types of cancers, and its expression level was found to be higher in cancer tissue compared to normal tissue. Therefore, the TCGA database was used to determine the differential expression of lncRNAs in LUAD. Figure 1A depicts the top 50 lncRNAs with aberrant expression in LUAD. The role of DARS-AS1 has not been investigated in depth in LUAD pathogenesis. As a result, DARS-AS1 was selected for the study. Based on the bioinformatics prediction from TCGA, DARS-AS1 was found to be overexpressed in LUAD compared to normal lung specimens (Figure 1B). Next, DARS-AS1 expression was determined in 50 pairs of LUAD patient specimens, with up-regulation of DARS-AS1 detected in LUAD tissues compared to adjacent normal specimens (Figure 1C, *p* < 0.001). QRT-PCR was conducted to further investigate the transcription level of DARS-AS1 in LUAD cell lines. The results revealed that DARS-AS1 was significantly up-regulated in LUAD cell lines (NCI-H23, A549, HCC827, PC-9, and C422L) when compared to normal bronchial epithelial cells (HBE) (Figure 1D, ^**^p < 0.01, ^*^p < 0.05). Among the five LUAD cell lines, A549 cells had the highest DARS-AS1 expression, while PC-9 had the lowest expression. Following that, the pathological association between DARS-AS1 and the overall survival rate of LUAD patients was analyzed using the Kaplan-Meier method. Based on the median of the DARS-AS1 expression levels, the patients were divided into a high expression group and a low expression group, with 25 cases in each group. The results indicated that LUAD patients with high DARS-AS1 expression (top 50%) had a shorter survival rate compared to those with low DARS-AS1 expression (bottom 50%). Furthermore, the five-year survival rate of patients with high DARS-AS1 expression was 21.3%, while it was 50.3% for patients with low DARS-AS1 expression (Figure 1E). These results collectively demonstrated that DARS-AS1 was upregulated in LUAD and correlated with poor survival in LUAD patients.

### LncRNA DARS-AS1 potentiated LUAD cell proliferative, migration, and invasion ability *in vitro*

**Figure 2:**
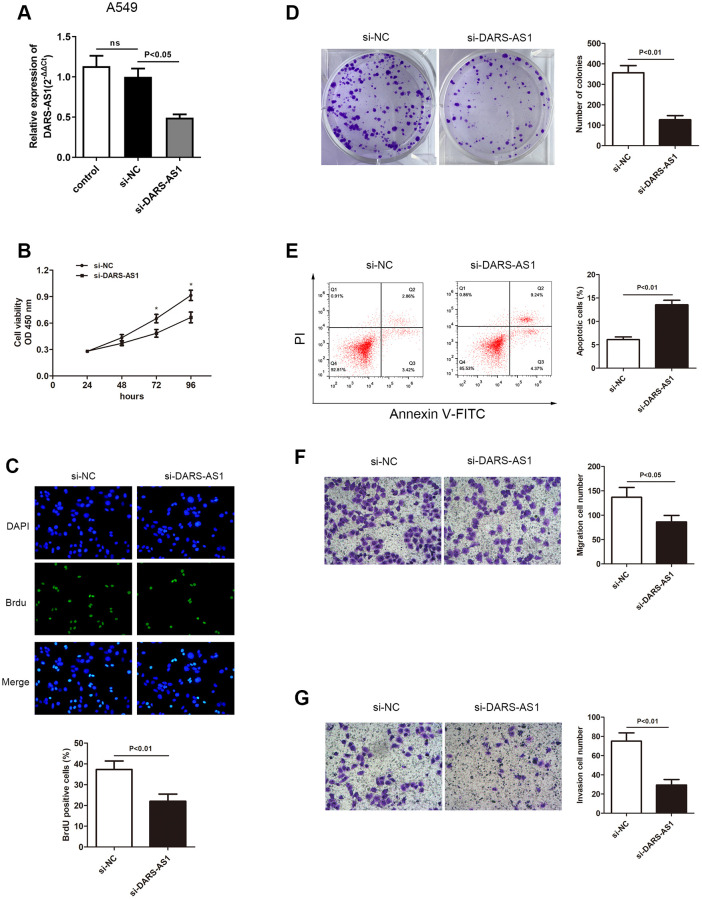
**Knockdown of DARS-AS1 resulted in alleviated LUAD progression.** (**A**) The DARS-AS1 expression level was significantly decreased in A549 cells compared to the si-NC group after transfection with si-DARS-AS1 (*p* < 0.05). DARS-AS1 knockdown resulted in a lower proliferation rate of LUAD cells according to the (**B**) CCK-8 (*p* < 0.05), (**C**) BrdU (*p* < 0.01), and (**D**) colony formation (*p* < 0.01) assays compared to the si-NC group. (**E**) An increased apoptosis rate was observed in the si-DARS-AS1 group compared to the si-NC group (upper right quadrant + lower right quadrant) (*p* < 0.01). A549 cells transfected with si-DARS-AS1 exhibited less (**F**) migrating (*p* < 0.05) and (**G**) invading (*p* < 0.01) cells compared to the si-NC group.

**Figure 3:**
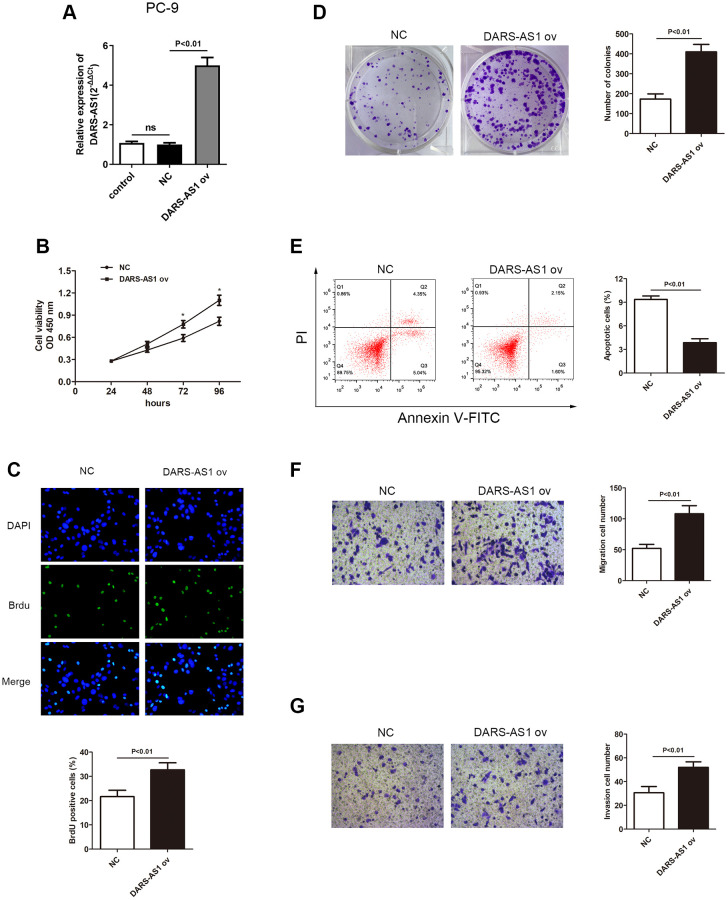
**Overexpression of DARS-AS1 resulted in accelerated LUAD progression.** (**A**) Expression of DARS-AS1 was significantly higher in PC-9 cells transfected with DARS-AS1 ov compared to the NC group (*p* < 0.01). (**B**) PC-9 cells with DARS-AS1 ov transfection exhibited greater proliferative capability according to the (**B**) CCK-8 (*p* < 0.05), (**C**) BrdU (*p* < 0.01), and (**D**) colony formation (*p* < 0.01) assays compared to the NC group. (**E**) Flow cytometry results showed a significantly lower apoptosis rate in the DARS-AS1 ov transfection group (upper right quadrant + lower right quadrant) compared to the NC group (*p* < 0.01). PC-9 with DARS-AS1 ov transfection exhibited a higher number of (**F**) migrating (*p* < 0.01) and (**G**) invading (*p* < 0.01) cells compared to the NC group.

The enhanced expression of DARS-AS1 in LUAD tissues and cell lines suggested that it could act as a cancer-promoting gene in LUAD. An investigation was conducted to validate this assumption. The DARS-AS1 overexpression and silencing systems were constructed using DARS-AS1 ov and si-DARS-AS1, respectively. The A549 cell line was transfected with si-DARS-AS1 and PC-9 cells were transfected with DARS-AS1 ov because expression of DARS-AS1 was highest in A549 cells and lowest in PC-9 cells based on the preceding assay. After 48 h of transfection, the transcription level of DARS-AS1 was detected by qRT-PCR. The results revealed significant DARS-AS1 down-regulation in the si-DARS-AS1 group (Figure 2A, *p* < 0.05) and overexpression in the DARS-AS1 ov group (Figure 3A, *p* < 0.01). In order to investigate the effect of DARS-AS1 on LUAD cell proliferation, the CCK-8, BrdU, and colony-forming assays were performed. The findings demonstrated that DARS-AS1 silencing induced a decrease in cell viability in A549 cells (Figure 2B, ^*^P < 0.05), while up-regulation of DARS-AS1 resulted in increased cell proliferation in PC-9 cells (Figure 3B, ^*^p < 0.05). Meanwhile, BrdU staining showed that there was less duplicated DNA (green) in the si-DARS-AS1 transfected group (Figure 2C, *p* < 0.01), while overexpression of DARS-AS1 led to increased cell replication (Figure 3C, *p* < 0.01), indicating that lncRNA DARS-AS1 overexpression was capable of enhancing LUAD cell proliferation. In addition, the results of colony formation assay of the si-DARS-AS1 group (Figure 2D, *p* < 0.01) were similar to the CCK-8 and BrdU assays, showing increased cell proliferation. The DARS-AS1 ov transfected group exhibited increased cell proliferation (Figure 3D, *p* < 0.01). The investigation of the DARS-AS1 mechanism in enhancing cell proliferation was performed using flow cytometry (FCM). The FCM demonstrated that the si-DARS-AS1 transfected group had an elevated cell apoptosis ratio (Figure 2E, *p* < 0.01), while up-regulation of DARS-AS1 reduced cell apoptosis (Figure 3E, *p* < 0.01), indicating that DARS-AS1 could enhance LUAD cell proliferation by inhibiting cell apoptosis. Subsequently, cell migration and invasion of LUAD with different expression levels of DARS-AS1 were detected using the transwell chamber assay. As shown in Figure 2F and 2G, the transwell assay revealed that silencing of DARS-AS1 resulted in attenuated cell migration and invasion ability (*p* < 0.01), while overexpression of DARS-AS1 in PC-9 cells led to enhanced cell migration and invasion (Figure 3F and 3G, *p* < 0.01). These results indicated that lncRNA DARS-AS1 was involved in promoting LUAD progress.

### LncRNA DARS-AS1 accelerated LUAD tumor development *in vivo*

**Figure 4:**
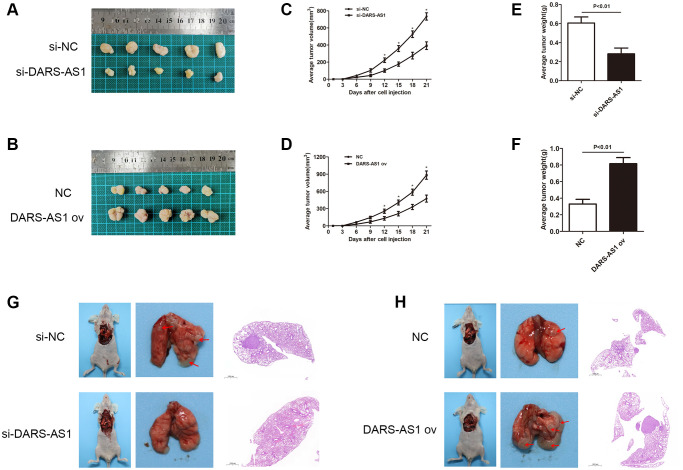
**DARS-AS1 promoted LUAD growth *in vivo*.** (**A**) LUAD transfected with si-DARS-AS1 had smaller tumors, while (**B**) the DARS-AS1 ov transfection group had larger tumors. The average tumor volume of LUAD transfected with (**C**) si-DARS-AS1 (*p* < 0.05 compared to si-NC) or (**D**) DARS-AS1 ov (*p* < 0.05 compared to NC group) was continuously measured over 21 days. After 21 days, the average tumor weight of LUAD transfected with (**E**) si-DARS-AS1 (*p* < 0.01) or (**F**) DARS-AS1 ov (*p* < 0.01) was scored. (**G**) The si-DARS-AS1 group had smaller tumors and fewer spreading lesions in the lung than the si-NC group. (**H**) The DARS-AS1 ov group had larger tumors and more spreading lesions in the lung than the NC group.

As LncRNA DARS-AS1 was confirmed as a cancer-promoting gene in LUAD *in vitro* through the above experiments, the effect of LUAD *in vivo* was examined through an animal study using nude mice. After injecting LUAD cells transfected with related plasmids (si-DARS-AS1, DARS-AS1 ov, and negative control) subcutaneously in the nude mice, the average tumor volume and weight were recorded. Figure 4A and 4B showed the LUAD tumor size in the si-DARS-AS1 or DARS-AS1 ov. groups. Consistent with the *in vitro* findings, knockdown of DARS-AS1 resulted in significantly smaller tumors compared to the negative control (Figure 4A), while DARS-AS1 overexpression resulted in bigger tumors (Figure 4B). Furthermore, DARS-AS1 silencing led to reduced tumor growth rate and weight (Figure 4C and 4E, ^*^p < 0.05), while opposite effects were observed with the up-regulation of DARS-AS1 (Figure 4D and 4F, ^*^p < 0.05).

The lung orthotopic tumor transplantation assay was used to assess the growth ability of tumor cells. At the sites of tumor cell injections, tumors developed in three out of five mice injected with A549 cells transfected with si-NC, and none in mice injected with A549 cells transfected with si-DARS-AS1. In addition, two mice injected with A549 cells transfected with si-NC had spreading lesions in the left and right lungs (Figure 4G and Table 1). Tumors developed in all five mice injected with PC-9 cells transfected with DARS-AS1 ov, and in two of five mice injected with PC-9 cells transfected with NC. Four mice injected with PC-9 cells transfected with DARS-AS1 ov had spreading lesions in the left and right lungs, while one mouse with PC-9 cells transfected with NC had spreading lesions. A comparison of tumor size at the injection site revealed that the DARS-AS1 ov group had significantly larger tumors than the NC group (Figure 4H and Table 1). The above *in vivo* experiments demonstrated that lncRNA DARS-AS1 was also capable of promoting LUAD development *in vivo*.

**Table 1:** Orthotopic tumor growth in nude mice.

**Cells**	**Orthotopic tumor formation** ***n* (%)**	**Spreading lesions formation** ***n* (%)**	**Tumor volume** **mm^3^**
si-DARS (*n* = 5)	0 (0%)	0 (0%)	0
si-NC (*n* = 5)	3 (60%)	2 (40%)	21.2 ± 19.7
DARS-AS1 ov (*n* = 5)	5 (100%)	4 (80%)	79 ± 15.2
NC (*n* = 5)	2 (40%)	1 (20%)	3.2 ± 4.6

### MiR-188-5p is a negatively regulatory target of DARS-AS1 and reversed LUAD promoting effect brought by DARS-AS1

**Figure 5:**
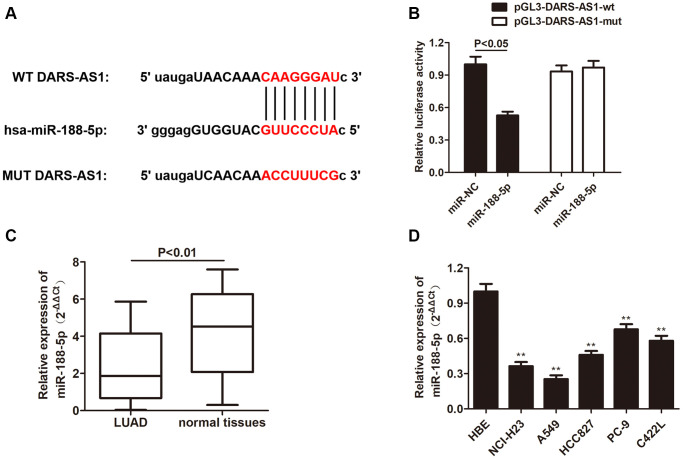
**MiR-188-5p is a target gene of DARS-AS1.** (**A**) The binding site between DARS-AS1 and miR-188-5p. (**B**) The dual-luciferase assay results confirmed a direct interaction between DARS-AS1 and miR-188-5p (*p* < 0.05). (**C**) The expression level of miR-188-5p was lower than usual in LUAD tissues compared to normal tissues (*p* < 0.01). (**D**) The expression level of miR-188-5p was lower than usual in LUAD cell lines compared to the HBE cell line (^**^p < 0.01).

Although DARS-AS1 has been identified as a cancer-promoting gene of LUAD, the exact regulatory pathway remains unknown. In order to investigate the downstream target of DARS-AS1, starBase was applied and there was an overlapping sequence between miR-188-5p and DARS-AS1 (Figure 5A), indicating a direct interaction between these two factors. Following that, the hypothesis was confirmed by dual-luciferase activity; miR-188-5p significantly reduced the luciferase activity in A549 cells transfected with pGL3- DARS-AS1-wt plasmid compared to the control group, while the luciferase activity in A549 cell transfected with pGL3-DARS-mut exhibited no changes (Figure 5B, *p* < 0.05). QRT-PCR was performed to determine the transcription level of miR-188-5p in LUAD tissues and cell lines. The results revealed a down-regulation of miR-188-5p in both LUAD tissues and cell lines compared to normal tissues and the HBE cell line, respectively (Figure 5C and 5D, ^**^p < 0.01), suggesting that aberrant low expression of miR-188-5p may have some pathological association with the development of LUAD.

**Figure 6:**
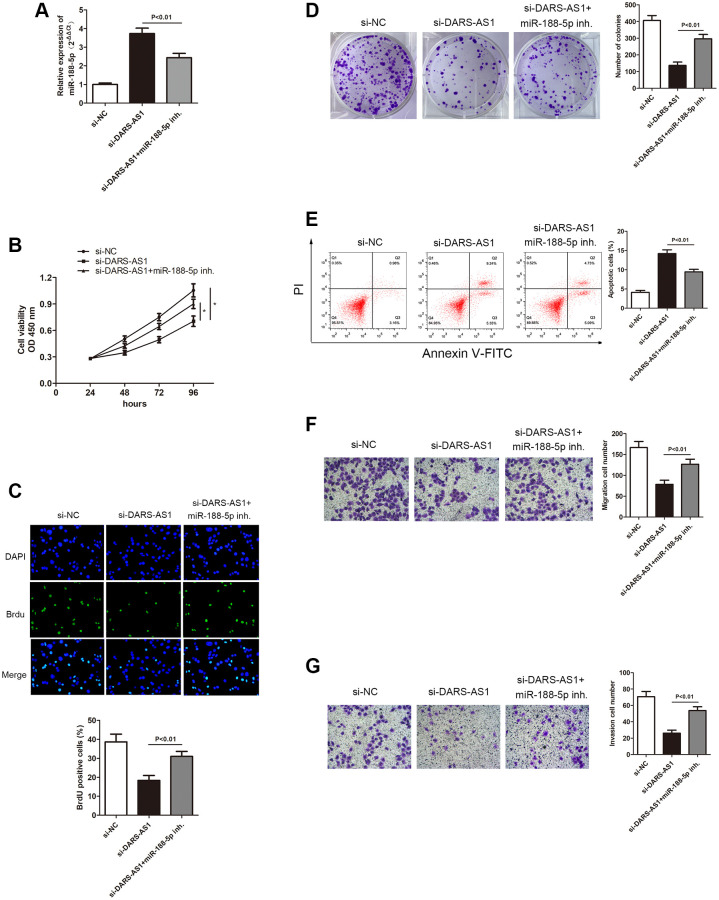
**Inhibition of miR-188-5p altered the suppression of A549 cell progression mediated by si-DARS-AS1.** (**A**) Three groups were transfected with si-DARS-AS1, si-DARS-AS1+miR-188-5p inhibitor, and si-NC in the A549 cell line. The cell proliferative capacity of the three groups was measured using the (**B**) CCK-8, (**C**) BrdU, and (**D**) colony formation assays (^*^p < 0.05). (**E**) Inhibition of miR-188-5p resulted in a significant reduction in the apoptosis ratio (upper right quadrant+lower right quadrant). The transwell chamber results showed that inhibition of miR-188-5p significantly facilitated cell migration (**F**) *p* < 0.01 and invasion (**G**) *p* < 0.01, which were suppressed by si-DARS-AS1.

In order to further investigate miR-188-5p in LUAD, we used DARS-AS1 knockdown to determine its expression level in LUAD. The qRT-PCR results demonstrated that DARS-AS1 silencing induced up-regulation of miR-188-5p. However, when the miR-188-5p inhibitor and si-DARS-AS1 were given simultaneously, there was a decrease in miR-188-5p expression (Figure 6A, *p* < 0.01), indicating that miR-188-5p was possible a negative target of DARS-AS1. The CCK-8, BrdU, and colony formation assays were used to determine the functional role of miR-188-5p in LUAD. Compared to the DARS-AS1 silencing group, co-treating with the miR-188-5p inhibitor and si-DARS-AS1 significantly enhanced cell viability (Figure 6B, ^*^p < 0.05). Similarly, findings from the BrdU and colony formation assays verified that knockdown of DARS-AS1 alone alleviated cell proliferation, but co-treating cells with the miR-188-5p inhibitor and si-DARS-AS1 improved cell viability (Figure 6C and 6D, *p* < 0.01). Flow cytometry and a transwell assay were also conducted to examine the effects of DARS-AS1 and miR-188-5p. According to FCM results, the miR-188-5p inhibitor and si-DARS-AS1 co-treated groups had a lower number of apoptotic cells compared to the solo si-DARS-AS1 group (Figure 6E, *p* < 0.01). In addition, the transwell assay was performed to determine the potentiated cell migration and invasive ability of cells that were given the miR-188-5p inhibitor and only si-DARS-AS1. The findings revealed that miR-188-5p inhibitor reserved the inhibition of A549 cell migration and invasion mediated by DARS-AS1 absence (Figure 6F and 6G, *p* < 0.01), indicating that miR-188-5p was a tumor suppressor in LUAD.

### MiR-188-5p alleviated LUAD development via inhibiting KLF12

**Figure 7:**
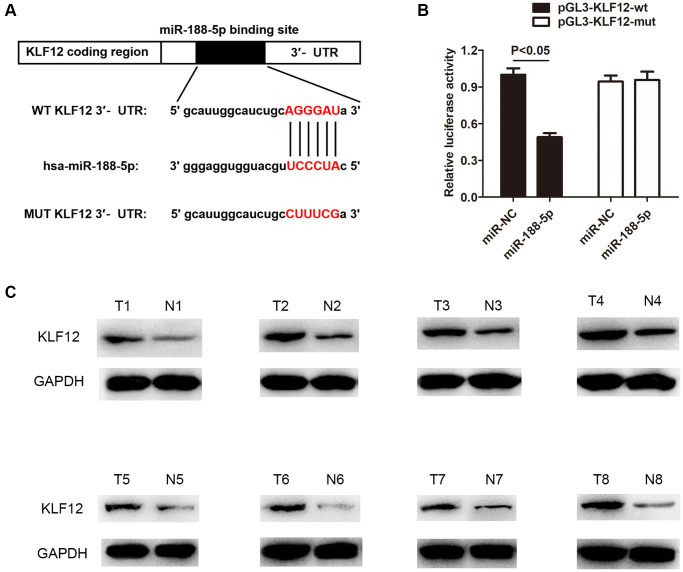
**KLF12 is a negative regulatory target of miR-188-5p.** (**A**) The binding site between KLF12 and miR-188-5p. (**B**) The dual-luciferase assay results confirmed the existence of direct interaction between KLF12 and miR-188-5p (*p* < 0.05). (**C**) High expression of KLF12 in LUAD tissues was detected by western blot.

Krüppel-like factor 12 (KLF12) is a transcription factor of the KLF family [27], which is mainly expressed as proteins. The KLF family 1–17 proteins are expressed in the human body [28]. Studies have shown that KLF12 protein can participate in abnormal proliferation and metastasis of tumor cells [29]. Nevertheless, there is still limited understanding regarding the function of KLF12 in LUAD. Therefore, we utilized starBase, TARGETMINER, and miRDB prediction to discover a binding site between miR-188-5p and KLF12 (Figure 7A). Next, the dual-luciferase activity assay demonstrated a direct interaction between KLF12 and miR-188-5p. Compared to the control group, miR-188-5p significantly reduced the luciferase activity in A549 cells transfected with the pGL3-KLF12-wt plasmid, while there was no change in luciferase activity in A549 cell transfected with pGL3-KLF12-mut (Figure 7B). In addition, western blot results indicated that the expression level of KLF12 was higher than usual in LUAD tissues (Figure 7C), indicating that KLF12 may play a role in potentiating the course of LUAD.

**Figure 8:**
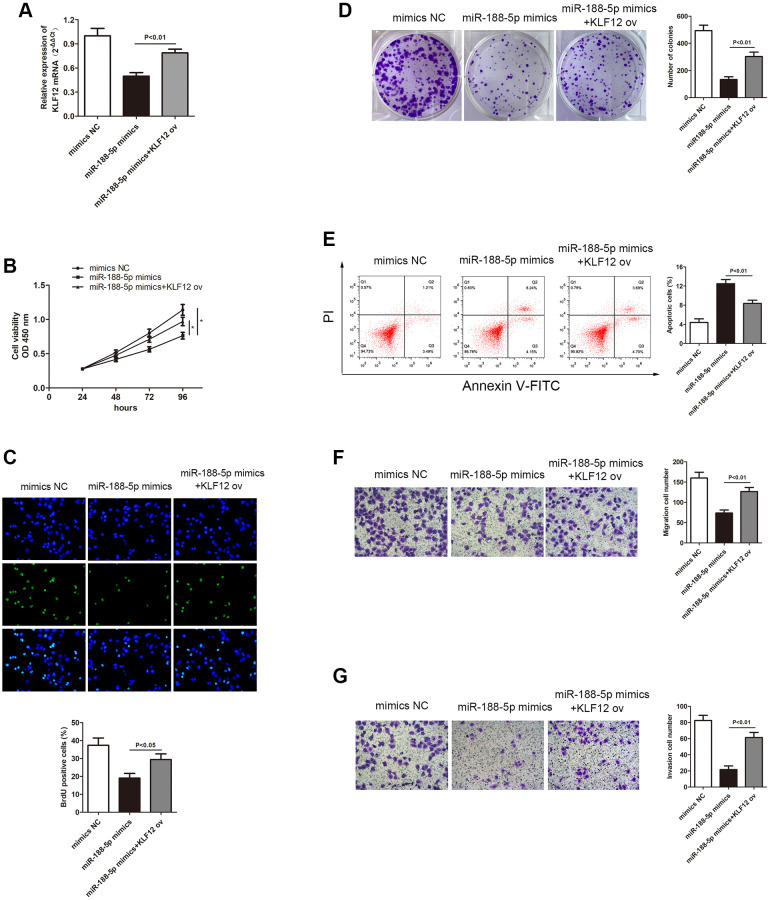
**Overexpression of KLF12 promoted LUAD progression.** (**A**) Three groups were transfected using miR-188-5p mimics, miR-188-5p mimics+KLF12 ov, and NC mimics in the A549 cell line. The cell proliferative capacity of the three groups was measured using the (**B**) CCK-8 (*p* < 0.05), (**C**) BrdU (^*^p < 0.05), and (**D**) colony formation (*p* < 0.01) assays. (**E**) Overexpression of KLF12 resulted in a significant reduction in the apoptosis ratio (upper right quadrant+lower right quadrant). The transwell chamber results indicated that overexpression of KLF12 significantly promoted cell (**F**) migration (*p* < 0.01) and (**G**) invasion (*p* < 0.01), which were suppressed by miR-188-5p.

In order to study the relationship between KLF12 and miR-188-5p, the transcription level of KLF12 in LUAD cells was measured using miR-188-5p mimics. The qRT-PCR results showed that overexpression of miR-188-5p led to KLF12 silencing, but giving KLF12 ov subsequently reversed the effect of the miR-188-5p mimics (Figure 8A, *p* < 0.01). This finding revealed that KLF12 is negatively regulated by miR-188-5p. The CCK-8, BrdU, and colony formation assays showed that overexpression of KLF12 could promote cell growth (Figure 8B–8D), indicating that KLF12 could reverse the suppressive effect of miR-188-5p on LUAD. Furthermore, we found that KLF12 ov reserved the inhibition of A549 cell migration and invasion mediated by miR-188-5p mimics from the transwell assay (Figure 8F and 8G, *p* < 0.01). Flow cytometry results revealed a significant reduction in apoptosis ratio in the KLF12 ov + miR-188-5p mimics group when compared to the solo miR-188-5p mimics group (Figure 8E, *p* < 0.01). These findings suggested that KLF12 could reverse the effects of miR-188-5p and mainly functions as a factor that facilitates the progression of LUAD development.

### DARS-AS1 conferred the LA course via stimulating the PI3K/AKT pathway

**Figure 9:**
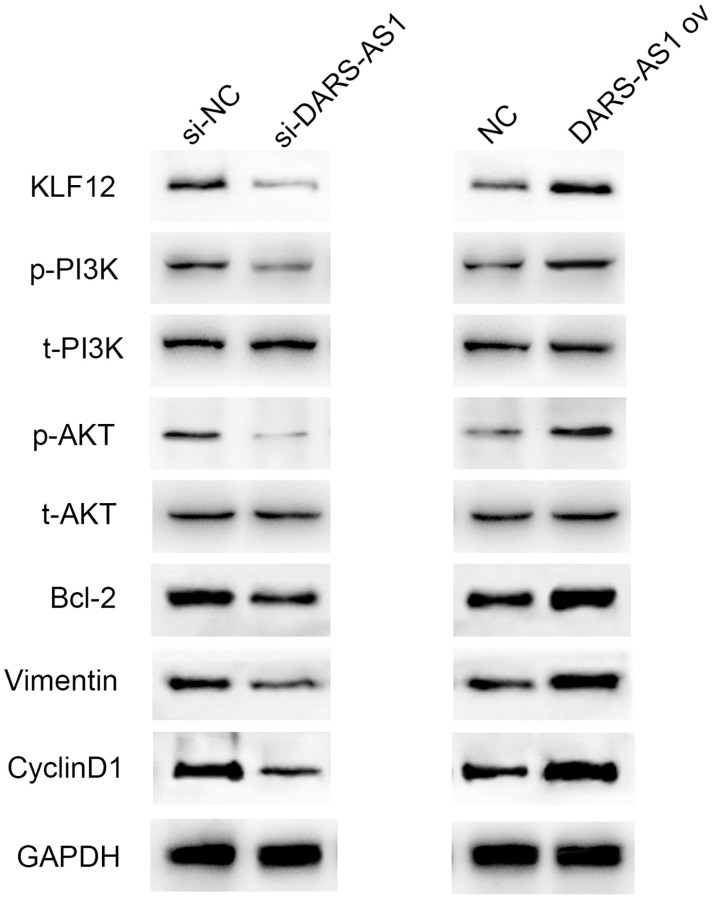
Western blot results demonstrated up-regulation of KLF12, p-PI3K, p-AKT, Bcl-2, Vimentin, and CyclinD1 in the DARS-AS1 overexpression group, while the proteins were down-regulated in the si-DARS-AS1 group.

In order to further understand the regulatory pathway of DARS-AS1, several typical tumorigenesis pathways were selected to investigate whether they were regulated by DARS-AS1. Figure 9 displays the western blot results, which demonstrated that overexpression of DARS-AS1 induced up-regulation of KLF12, p-PI3K, p-AKT, Bcl-2, Vimentin, and CyclinD1, while DARS-AS1 silencing had the opposite effect. These findings confirmed that DARS-AS1 promoted the development of LUAD via activation of the PI3K/AKT pathway, inhibited cell apoptosis by up-regulating Bcl-2, and accelerated the EMT and cell cycle processes by overexpressing Vimentin and CyclinD1.

## DISCUSSION

Lung adenocarcinoma has been ranked as the fifth leading cause of cancer deaths worldwide. Due to the nonspecific early symptoms, the majority of the diagnosed LUAD patients are in the middle and late stages, with multiple metastases, and have missed the optimal period for treatment [30]. In recent years, the development of gene sequencing technology has increased our understanding of the molecular pathogenesis of lung cancer. This promotes the generation of new molecular markers, the development of targeted therapy, and improves the overall survival of patients with advanced or metastatic diseases [31].

Long non-coding RNAs (lncRNAs) are a type of long-chain RNA molecules that do not participate in protein-coding *in vivo*. It has been reported that lncRNAs play an important role in the occurrence and development of tumors. LncRNA DARS-AS1 has been identified as a tumor facilitator in both clear cell renal cell carcinoma [13], ovarian cancer [12], and cervical cancer [32]. However, there is still limited understanding of the exactly pathological correlation between DARS-AS1 and LUAD. This is the first study that identified lncRNA DARS-AS1 as a cancer-promoting gene in LUAD, with higher-than-usual expression content of DARS-AS1 detected in LUAD tissues and cell lines. Furthermore, the cell functional experiments of DARS-AS1 were performed using si-DARS-AS1 and DARS-AS1 ov in LUAD cells to examine the effect of DARS-AS1 on LUAD. The results showed that up-regulation of DARS-AS1 induced potentiated LUAD cell growth via inhibition of apoptosis, as well as augmented cell migration and invasion. As DARS-AS1 silencing resulted in the opposite effect, it validated our hypothesis that DARS-AS1 conferred LUAD with stronger cell growth, migration, and invasion.

Mir-188-5p is a tumor therapeutic factor in prostate cancer [33], cytogenetically normal acute myeloid leukemia [20], gastric cancer [34], oral squamous cell carcinoma [35], and others. However, further investigation into the role of miR-188-5p in LUAD is required. Using bioinformatics analysis, it was discovered that miR-188-5p may be involved in modulating LUAD progression by interacting with DARS-AS1. Furthermore, miR-188-5p was found to be a negatively regulated target gene of DARS-AS1 through the dual-luciferase assay, and aberrant down-regulation of miR-188-5p was detected in LUAD tissues and cell lines. Knockdown of DARS-AS1 resulted in increased transcription of miR-188-5p. In addition, inhibition of miR-188-5p resulted in enhanced proliferation, migration, and invasion. There has been much debate over the exact role of miR-188-5p in tumors. A study found that miR-188-5p suppressed cancer cell growth in acute promyelocytic leukemia [36], while another discovered that miR-188-5p could inhibit cell proliferation by potentiating apoptosis [37]. In this study, we discovered that miR-188-5p was a downstream target gene of lncRNA DARS-AS1 and inhibited LUAD development.

Krüppel-like factor 12 (KLF12) is a eukaryotic transcription factor that is involved in many biological processes, such as organ development, lipid metabolism, decidualization of endometrial stromal cells, proliferation, invasion, and anoikis of tumor cells. In colon cancer, KLF12 promotes the growth of colorectal cancer through EGR1 (early growth response protein 1) [38]. Furthermore, a previous study reported that KLF12 protein is highly expressed in cervical cancer cells. However, there is limited research on KLF12 in NSCLC, especially LUAD. In this study, we discovered for the first time that KLF12 functioned as an enhancing factor in the development of LUAD. In addition, the modulatory axis that DARS-AS1 conferred LUAD development via the miR-188-5p/KLF12 axis was evident. The regulatory pathways that were involved included PI3K/AKT, EMT, apoptosis, and cell cycle process. These findings establish for the first time that DARS-AS1/miR-188-5p/KLF12 is a newly discovered regulatory axis that may offer a novel curative strategy for early diagnosis and treatment of LUAD.
